# Degradation of the Incretin Hormone Glucagon-Like Peptide-1 (GLP-1) by Enterococcus faecalis Metalloprotease GelE

**DOI:** 10.1128/mSphere.00585-19

**Published:** 2020-02-12

**Authors:** Stephanie L. LeValley, Catherine Tomaro-Duchesneau, Robert A. Britton

**Affiliations:** a Department of Molecular Virology and Microbiology, Baylor College of Medicine, Houston, Texas, USA; University of California, Davis

**Keywords:** *Enterococcus*, GLP-1, GelE, gut hormone, protease

## Abstract

Metabolic diseases, including type 2 diabetes and obesity, have become increasingly prevalent global health concerns. Studies over the past decade have established connections between the gastrointestinal microbiota and host metabolism, but the mechanisms behind these connections are only beginning to be understood. We were interested in identifying microbes that have the ability to modulate the levels of the incretin hormone glucagon-like peptide-1 (GLP-1). Using a human-derived cell line that is capable of secreting GLP-1 in response to stimulatory ligands (NCI-H716), we identified supernatants from several bacterial isolates that were capable of decreasing GLP-1 levels, including several strains of Enterococcus faecalis. We further identified the secreted protease GelE, an established virulence factor from E. faecalis, as being responsible for GLP-1 inhibition via direct cleavage of GLP-1 by GelE. Finally, we demonstrated that E. faecalis supernatants can disrupt a colonic epithelial monolayer and cleave GLP-1 in a *gelE*-dependent manner. This work suggests that a secreted factor from an intestinal microbe can traverse the epithelial barrier and impact levels of an important intestinal hormone.

**IMPORTANCE** Humans have a complex and interconnected relationship with their gastrointestinal microbiomes, yet our interest in the microbiome tends to focus on overt pathogenic or probiotic activities, leaving the roles that commensal species may have on host physiology and metabolic processes largely unexplored. Commensal organisms in the microbiome produce and secrete many factors that have an opportunity to interact with the gastrointestinal tract and host biology. Here, we show that a secreted protease from E. faecalis, GelE, is able to degrade the gastrointestinal hormone GLP-1, which is responsible for regulating glucose homeostasis and appetite in the body. The disruption of natural GLP-1 signaling by GelE may have significant consequences for maintaining healthy blood glucose levels and in the development of metabolic disease. Furthermore, this work deepens our understanding of specific host-microbiome interactions.

## Abstract (precis)

Humans have a complex and interconnected relationship with their gastrointestinal microbiomes, yet our interest in the microbiome tends to focus on overt pathogenic or probiotic activities, leaving the roles that commensal species may have on host physiology and metabolic processes largely unexplored. Commensal organisms in the microbiome produce and secrete many factors that have an opportunity to interact with the gastrointestinal tract and host biology. Here, we show that a secreted protease from E. faecalis, GelE, is able to degrade the gastrointestinal hormone GLP-1, which is responsible for regulating glucose homeostasis and appetite in the body. The disruption of natural GLP-1 signaling by GelE may have significant consequences for maintaining healthy blood glucose levels and in the development of metabolic disease. Furthermore, this work deepens our understanding of specific host-microbiome interactions.

## INTRODUCTION

The human gastrointestinal (GI) tract is home to trillions of microorganisms, collectively referred to as the GI microbiome (1). Because of the direct physical proximity that the gut microbiome has with its human host, it is no surprise that the microbiome plays a role in multiple aspects of health, the best characterized of these being immune tolerance, pathogen resistance, and digestion (2). Less understood are the interactions between the microbiome and human metabolism. Despite limited mechanistic insight into the cross-section of microbiome and host metabolism, it is of great interest both as an etiology of disease and for potential therapeutic applications, and some understanding is beginning to emerge. The first insights into microbial influence over human metabolism came from studies demonstrating that the simple absence of a microbiome resulted in decreased total body fat in germfree mice compared to that of conventional mice, independent of food intake; further, the decrease in fat mass could be gained back by colonizing the germfree mice with bacterial communities from conventionally raised mice (3). The observed ability of the microbiome to help harvest energy from the diet sparked a variety of research studies over the next decade, with a focus on the interaction of the microbiome with GI hormone peptides, in particular the nutrient-stimulated incretin glucagon-like peptide-1 (GLP-1). Already a therapeutic target for type 2 diabetes (T2D), GLP-1 is an integral signaling hormone responsible for promoting insulin secretion and satiety while decreasing glucagon secretion and gastric emptying. Early work showed that the addition of the prebiotic oligofructose to the diets of rats on a high-fat diet increased GLP-1 levels measured from the portal vein, and it protected them from weight gain (4). Additional studies demonstrated that the administration of Akkermansia muciniphila could reverse high-fat-diet-induced metabolic disorders and that this activity was mediated at least partially by an outer membrane protein purified from *A. muciniphila* interacting with Toll-like receptor 2 (5, 6). These studies strikingly demonstrate that a specific bacterial species and factor are capable of impacting metabolic disease phenotypes. Despite a heightened research interest, additional mechanisms behind how the microbiome and host metabolism influence each other remain largely undescribed.

Some of the obvious suspects to investigate for host-microbiome interactions are the many secreted proteins and metabolites that bacteria release as part of their natural life cycle. While these external products are often part of bacterial cellular metabolism or provide beneficial functions to the bacterial cell, they also have an opportunity to interact with their environment, in this case, the human GI tract. Here, our screening for bacterial modulators of GLP-1 revealed multiple bacterial strains that can inhibit GLP-1 levels in an *in vitro* assay. We further characterize this inhibition as direct cleavage from E. faecalis strains by its secreted protease GelE, revealing a novel cleavage target of GelE. Finally, we suggest a role for GelE in disrupting natural GLP-1 signaling and metabolic processes.

## RESULTS

### Screening a human-derived bacterial library for GLP-1 modulatory activity.

**FIG 1:**
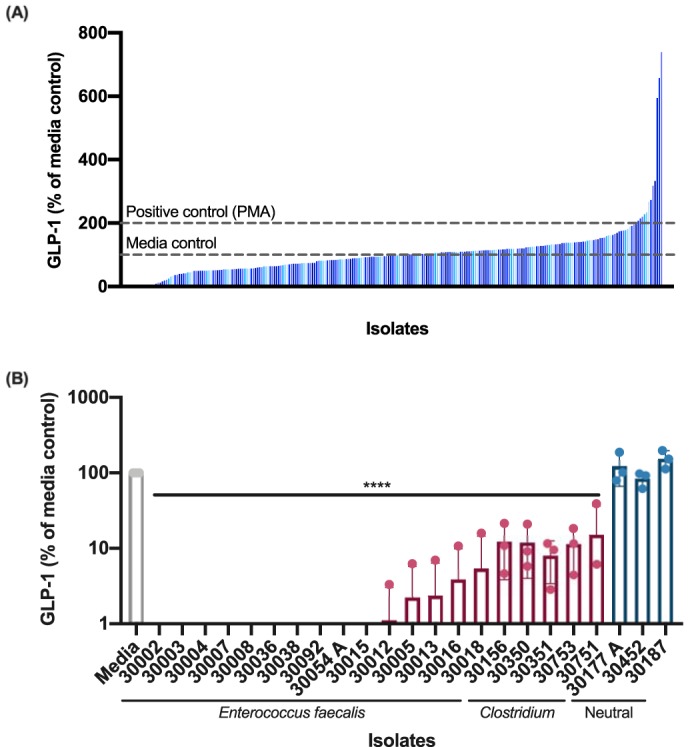
Screening for modulation of GLP-1. (A) Bacterial supernatants have a wide range of effects on GLP-1 secretion after incubation with NCI-H716 cells. Blue vertical bars represent individual bacterial isolates. Dashed horizontal lines represent GLP-1 secretion levels compared to the medium controls (100%) and a positive control, PMA (200%). (B) GLP-1-inhibitory isolates. Multiple isolates of Enterococcus faecalis and *Clostridium* decreased GLP-1 levels compared to the medium control. Data were obtained from three independent experiments (*n* = 3) and are expressed as the mean ± standard deviation (SD) values (****, *P* < 0.0001 with one-way ANOVA).

Bacterial strains capable of modulating GLP-1 levels were identified by an *in vitro* screening pipeline using the GLP-1-secreting human cell line NCI-H716 (7). Over 1,500 cell supernatants collected from individual bacterial isolates were prepared and applied to NCI-H716 cell monolayers for 2 h, and the secretion of GLP-1 into the medium was measured with an enzyme-linked immunosorbent assay (ELISA). NCI-H716 cell viability was also monitored by the PrestoBlue cell viability reagent to ensure no significant increase in NCI-H716 cell lysis or death (data not shown). The majority of the bacterial isolates screened had no impact on GLP-1 levels; however, approximately 20 isolates showed a marked decrease in GLP-1 levels, many of them below the limit of detection of the ELISA (Fig. 1A). We also identified 45 isolates that dramatically increase GLP-1 levels; these were further characterized in a separate study (8).

To identify the species of each isolate, the 16S rRNA gene was sequenced. The majority of the isolates identified as Enterococcus faecalis, as well as Clostridium perfringens, Clostridium bifermentans, and Clostridium butyricum. The E. faecalis isolates exhibited a stronger GLP-1-inhibitory effect, ranging from 0% ± 0.0% to 5.44% ± 9.1% GLP-1 compared to the medium controls (*P < *0.0001, Fig. 1B). The *Clostridium* species, while still inhibitory, consistently show a slightly weaker inhibitory effect, ranging from 8.0% ± 4.6% to 15.2% ± 21.0% GLP-1 compared to the medium controls (*P < *0.0001, Fig. 1B). Because of this difference in the GLP-1-inhibitory activities of E. faecalis and *Clostridium* species, we decided to further characterize the activities of the E. faecalis isolates.

### Identifying the factor secreted from E. faecalis responsible for GLP-1 inhibition.

**FIG 2:**
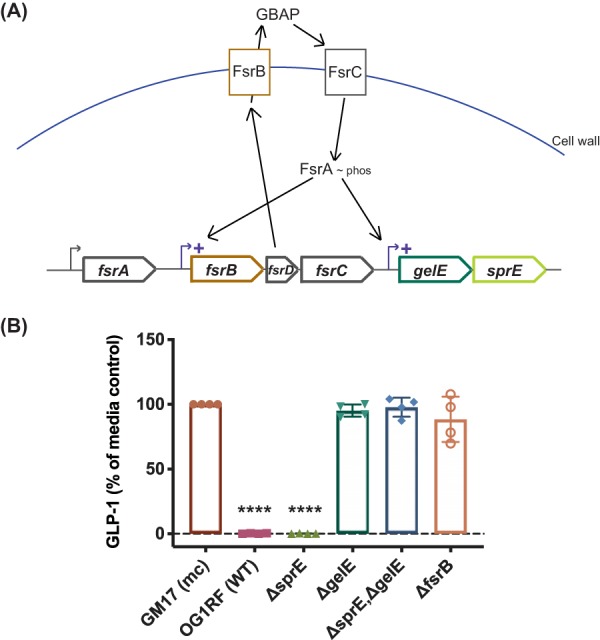
Identifying the factor responsible for GLP-1 inhibition. (A) Genetic pathway for the production of E. faecalis protease-encoding genes *gelE* and *sprE*. Activation of the quorum sensing system *fsr* activates the transcription of *gelE* and *sprE* immediately downstream of the *fsr* operon. (B) E. faecalis strains unable to produce a functional GelE no longer deplete GLP-1 levels in an *in vitro* coincubation of bacterial supernatants with GLP-1. Data were obtained from four independent experiments (*n* = 4) and are expressed as the mean ± SD [****, *P* < 0.0001 compared to GM17 (mc) with one-way ANOVA]. GM17 (mc), bacterial culture medium control.

Size fractionation experiments showed that GLP-1-inhibitory activity from E. faecalis was contained within the 30- to 50-kDa size fraction of the supernatant (see Fig. S1 in the supplemental material). We also found that the GLP-1 degradation activity was sensitive to heat and the metal ion chelator *N,N,N′,N′*-tetrakis(2-pyridylmethyl)ethane-1,2-diamine (TPEN) (Fig. S2 and S3). These data suggest that the factor responsible for GLP-1-inhibitory activity is a secreted, metal-dependent protein. Two secreted proteases from E. faecalis are well known, the serine protease SprE and the metalloprotease GelE, both of which are regulated by the two-component, quorum-sensing *fsr* (*faecalis* system regulator) operon (9) (Fig. 2A). We obtained strains of E. faecalis containing null mutations in *sprE* (TX5243), *gelE* (TX5264), and *fsrB* (TX5266), as well as a *gelE sprE* double mutant (TX5128), all generated in the commonly used wild-type OG1RF E. faecalis strain (10). To test the GLP-1-inhibitory activity of these strains, supernatants were incubated directly with recombinant GLP-1 (Tocris) (Fig. 2B). Of the strains tested, only the Δ*sprE* mutant strain maintained GLP-1-inhibitory activity equal to that of wild-type OG1RF (OG1RF, −0.06% ± 0.76%; Δ*sprE* mutant, −0.14% ± 0.74%, *P < *0.0001), while the Δ*gelE*, Δ*fsrB*, and Δ*gelE* Δ*sprE* mutant strains no longer showed decreased GLP-1 levels (95.19% ± 4.48%, 97.8% ± 7.37%, and 88.41% ± 17.47%, respectively). Taken together, the loss of GelE, either directly by knockout or indirectly by dysregulation through FsrB, results in a loss of GLP-1-inhibitory activity, suggesting cleavage of GLP-1 by the metalloprotease GelE.

10.1128/mSphere.00585-19.1 FIG S1 Sensitivity of GLP-1-inhibitory activity to TPEN. Supernatants from E. faecalis 30054A grown overnight with increasing amounts of metalloprotease inhibitor TPEN (higher specificity for zinc) result in a reduction in GLP-1 degradation when incubated with recombinant GLP-1, in a dose-dependent manner. Data were obtained from three (*n* = 3) independent experiments (replicates 1 to 3) with mean ± SD shown for each replicate (technical variation). Linear regression with *R*^2^ goodness-of-fit value and *P* value are reported. Download FIG S1, EPS file, 0.1 MB. Copyright © 2020 LeValley et al. 2020 LeValley et al. This content is distributed under the terms of the Creative Commons Attribution 4.0 International license.

10.1128/mSphere.00585-19.2 FIG S2 Size fractionation of E. faecalis supernatants to determine the size of the molecule responsible for GLP-1-inhibitory activity. Supernatants from E. faecalis 30054A were fractioned using centrifugal filters of various kilodalton cutoffs and then incubated on NCI-H716 cells. GLP-1-inhibitory activity is maintained in all fractions of >30 kDa. Data were obtained from one independent experiment (*n* = 1). Download FIG S2, EPS file, 0.1 MB. Copyright © 2020 LeValley et al. 2020 LeValley et al. This content is distributed under the terms of the Creative Commons Attribution 4.0 International license.

10.1128/mSphere.00585-19.3 FIG S3 GLP-1-inhibitory activity after heat treatment. Supernatants and whole-cell lysates from E. faecalis 30054A were treated for 30 min at 100°C, resulting in a reduction of GLP-1-inhibitory activity when incubated with NCI-H716 cells. GLP-1-inhibitory activity is primarily contained in the bacterial supernatant as opposed to the cellular lysate. Data were obtained from three independent experiments (*n* = 3) and are expressed as the mean ± SD. Download FIG S3, EPS file, 0.1 MB. Copyright © 2020 LeValley et al. 2020 LeValley et al. This content is distributed under the terms of the Creative Commons Attribution 4.0 International license.

**FIG 3:**
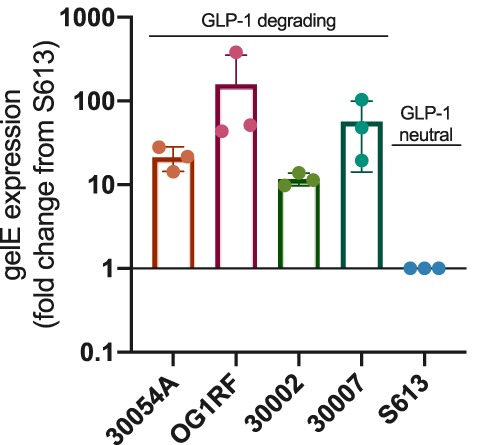
Expression of *gelE* and correlation with GLP-1 degradation. GLP-1-degrading strains of E. faecalis have significantly higher expression levels of *gelE* as measured by qPCR than does GLP-1-neutral strain S613. Data were obtained from three independent experiments (*n* = 3) and are expressed as the mean ± SD.

To further characterize GLP-1 cleavage by GelE, the expression of *gelE* in five E. faecalis isolates was measured by quantitative PCR in relation to their ability to cleave GLP-1 (Fig. 3). During the initial screen, we identified one E. faecalis isolate that did not inhibit GLP-1, the clinical isolate E. faecalis S613 (from Cesar A. Arias’s laboratory, University of Texas, Health Science Center) (11). The four GLP-1-degrading isolates highly express *gelE*, with expression levels at least 10-fold higher and up to 380-fold higher than those of the non-GLP-1-degrading isolate S613, demonstrating a correlation between *gelE* expression and GLP-1 degradation.

Throughout this study, our primary measurement of GLP-1 has been by ELISA, making it important to ensure that the decreases in GLP-1 we observe are indeed from the degradation of GLP-1 and not interference in the ELISA. Knowing that degradation activity is lost after prolonged exposure to heat (S3), we heat treated E. faecalis supernatants before and after incubation with GLP-1. If GelE was targeting the ELISA and not GLP-1, one would expect to see high levels of GLP-1 in a sample heat treated after incubation with GLP-1, ensuring no further degradation by GelE during the ELISA. Instead, we observe low GLP-1 levels from E. faecalis 30054A and 30002 supernatants heat treated after incubation with GLP-1 (Fig. S4), showing that degradation took place prior to heat treatment during the GelE–GLP-1 incubation.

10.1128/mSphere.00585-19.4 FIG S4 GLP-1-inhibitory activity with heat treatment pre- and postincubation. Supernatants from E. faecalis 30054A and 30002 were treated for 30 min at 100°C either before or after being incubated with 200 pM GLP-1. Data were obtained from two independent experiments (*n* = 2) and are expressed as the mean ± SD. Download FIG S4, PDF file, 0.02 MB. Copyright © 2020 LeValley et al. 2020 LeValley et al. This content is distributed under the terms of the Creative Commons Attribution 4.0 International license.

### GelE specificity for human metabolic substrates.

**FIG 4:**
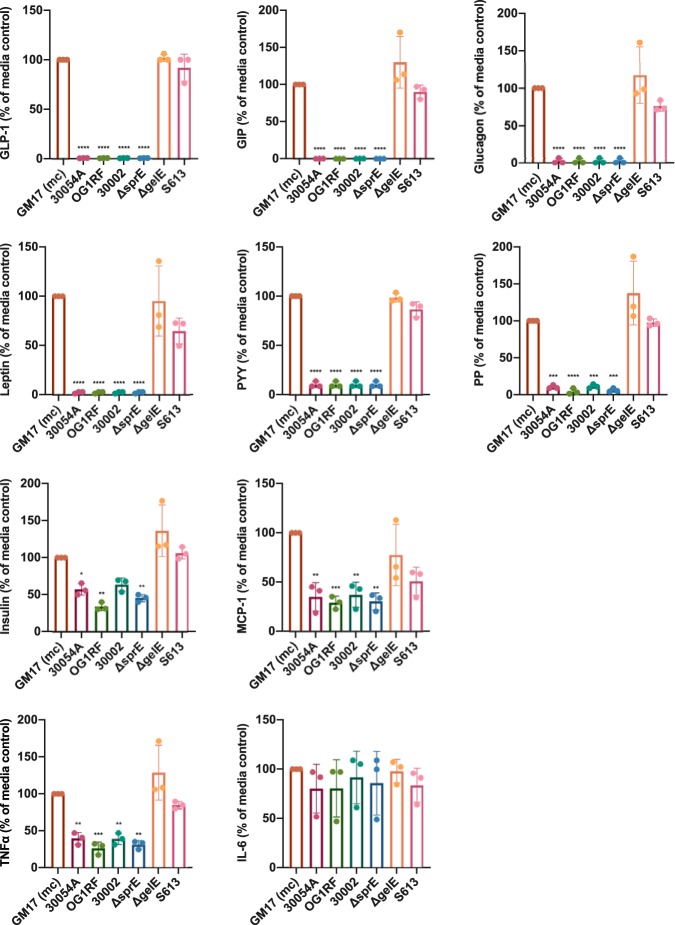
GelE specificity for human metabolic substrates. GelE is capable of degrading many human metabolic substrates, as measured by substrate concentration using a Luminex assay after incubation with E. faecalis supernatants. Data were obtained from three independent experiments (*n* = 3) and are expressed as the mean ± SD. For GLP-1, GIP, glucagon, leptin, PYY, and PP: ****, *P* < 0.0001. For insulin: 30054A *, *P* = 0.0351; OG1RF **, *P* = 0.001; Δ*sprE* **, *P* = 0.0058. For MCP-1: 30054A **, *P* = 0.0021; OG1RF ***, *P* = 0.0009; 30002 **, *P* = 0.0029; Δ*sprE* **, *P* = 0.0011. For TNF-α: 30054A **, *P* = 0.0038; OG1RF ***, *P* = 0.0006; 30002 **, *P* = 0.0036; Δ*sprE* **, *P* = 0.0012 with one-way ANOVA. GM17 (mc), bacterial culture medium control.

Previously, it has been shown that GelE degrades a range of substrates (12); thus, we wanted to better understand the range and specificity of GelE cleavage targets relevant to GLP-1 and other proteins involved in human metabolism. Supernatants from E. faecalis 30054A, 30002, OG1RF, Δ*gelE* mutant, Δ*sprE* mutant, and S613 strains were incubated with a panel of recombinant protein substrates (GLP-1, gastric inhibitory peptide/glucose-dependent insulinotropic peptide [GIP], peptide YY [PYY], leptin, glucagon, pancreatic peptide, insulin, interleukin 6 [IL-6], tumor necrosis factor alpha [TNF-α], and monocyte chemoattractant protein-1 [MCP-1]) and quantified by a Luminex assay. From our findings, it appears that GelE is capable of degrading, to some extent, nearly all of the substrates tested in the metabolic panel (Fig. 4); however, some patterns of degradation emerged. Recapitulating our previous findings, GLP-1 levels were reduced to 0.57% ± 0.13%, down to the limit of detection of the assay. Similar degradation was observed for GIP, glucagon, leptin, PYY, and pancreatic peptide. For insulin, MCP-1, and TNF-α, degradation was less striking but still statistically significant. Finally, we did not observe consistent degradation of IL-6 by GelE.

To gain a better understanding of the substrate preference of GelE, we also tested the ability of diluted supernatants of E. faecalis 30054A, from 1 to 0.0001×, to degrade the same panel of metabolic substrates (Table 1). Most prominently, supernatant from E. faecalis 30054A diluted to 0.01× still degraded the majority of GLP-1 present, leaving only 10.38% ± 3.52% GLP-1 remaining. GIP, glucagon, and leptin also show high degradation with diluted supernatants (1.55% ± 1.09%, 3.08% ± 3.25%, and 7.95% ± 4.72% substrate remaining, respectively, for 0.1× supernatants). This level of degradation does not hold true for all substrates tested, as pancreatic peptide and PYY maintain fairly high levels of degradation with undiluted supernatants (10.35% ± 2.4% and 10.45% ± 3.1% substrate remaining, respectively, for undiluted supernatants), but degradation lessens upon dilution (47.49% ± 17.15% and 54.61% ± 13.14% substrate remaining, respectively, for 0.1× supernatants). Finally, MCP-1, TNF-α, and insulin show less degradation even with undiluted supernatants (35.14% ± 14.1%, 39.73% ± 8.0%, and 57.29% ± 7.7% substrate remaining, respectively, for undiluted supernatants).

**TABLE 1:**
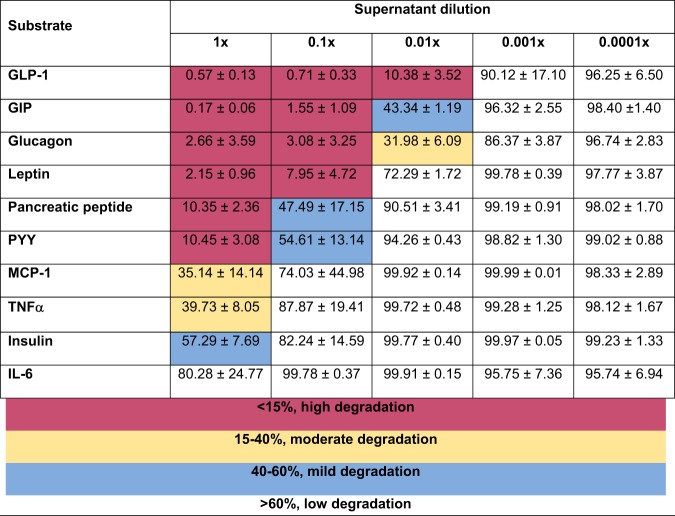
Degradation of metabolic substrates by E. faecalis 30054A diluted supernatant^*a*^

a Dilutions of supernatant from E. faecalis 30054A were incubated with various metabolic substrates, followed by measurement of substrate concentrations using a Luminex assay. Supernatant dilutions show an uneven distribution of degradation. Values represent the quantity (%) of substrate remaining after incubation with supernatants. Data were obtained from three independent experiments (*n* = 3) and are expressed as the mean ± standard deviation (SD). Less than 15% indicates high degradation, 15 to 40% indicates moderate degradation, 40 to 60% indicates mild degradation, and >60% indicates low degradation.

### Interaction of GelE and GLP-1 through an epithelial layer.

**FIG 5:**
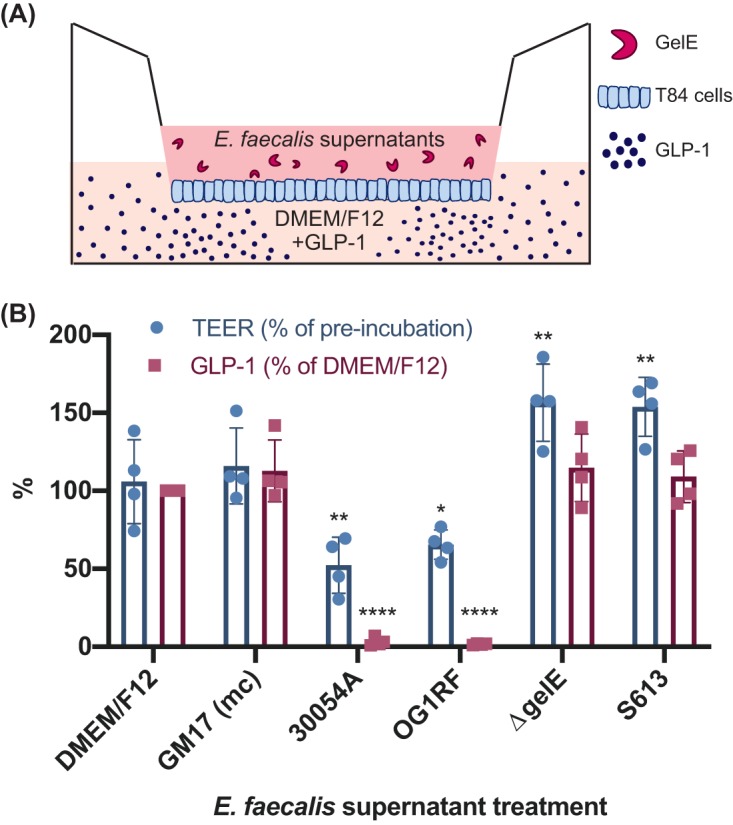
Impact of apical E. faecalis supernatants on basolateral GLP-1 degradation. (A) Schematic of experimental setup. T84 epithelial cells seeded onto a transwell insert, with E. faecalis supernatants added to the upper chamber and GLP-1 in cell culture medium (DMEM–F-12) to the lower chamber (schematic not drawn to scale). (B) After incubation, GelE-producing supernatants decrease the integrity of a T84 epithelial cell layer while degrading GLP-1 on the basolateral side of the epithelium, compared to the DMEM–F-12 T84 cell culture medium control. Data were obtained from four independent experiments and are expressed as the mean ± SD. For TEER: 30054A **, *P* = 0.0022; OG1RF *, *P* = 0.0439; Δ*gelE* **, *P* = 0.0045; S613 **, *P* = 0.0084. For GLP-1: 30054A and OG1RF ****, *P* < 0.0001 with two-way ANOVA. GM17 (mc), bacterial culture medium control.

While GelE from E. faecalis may readily cleave GLP-1 and other substrates *in vitro*, it is important to consider the GI epithelium that separates these two molecules *in vivo*. Previous work has implicated E. faecalis, and specifically GelE, in contributing to intestinal epithelium disruption (13–15). We aimed to model the ability of GelE to contact GLP-1 through an epithelial layer using T84 epithelial cells in a transwell format to mimic the microbial interface on the apical side and the presence of GLP-1 on the basolateral side of the epithelium (Fig. 5A). The integrity of the T84 epithelial layer, as measured by transepithelial electrical resistance, decreased by approximately half with the apical addition of cell-free E. faecalis supernatants expressing GelE from the 30054A and OG1RF strains (52.2% ± 17.97%, *P* = 0.0022, and 65.5% ± 9.47%, *P* = 0.0439, respectively, compared to the DMEM–F-12 T84 cell culture medium control), while E. faecalis supernatants lacking GelE, i.e., the Δ*gelE* mutant and S613 strains, increased the integrity of the epithelial layer (156.6% ± 24.76%, *P* = 0.0045, and 153.7% ± 18.93%, *P* = 0.0084, respectively, compared to the DMEM–F-12 T84 cell culture medium control) (Fig. 5B).

The basolateral compartment of each transwell contained approximately 500 pM GLP-1 supplemented into the DMEM–F-12 medium. When GelE containing supernatants 30054A or OG1RF was added apically to the T84 cell epithelial layer, GLP-1 in the basolateral compartment was cleaved nearly completely (3.1% ± 2.68%, *P* < 0.0001, and 1.6% ± 0.46%, *P* < 0.0001, respectively, compared to DMEM–F-12 T84 cell culture medium control), while GLP-1 in the basolateral compartment of apical supernatants from E. faecalis Δ*gelE* mutant and S613 strains remained intact (Fig. 5B). Together, these data demonstrate that GelE causes moderate damage to an epithelial layer, allowing access to the basolateral side where it can cleave GLP-1.

## DISCUSSION

Bacterial cells secrete a wide range of proteins and metabolites during their life cycle, and one such class of secreted molecules are proteases, enzymes that break down other proteins or peptides. A well-studied example of this is Enterococcus faecalis and its gelatinase GelE. The secreted metalloprotease GelE serves E. faecalis by degrading misfolded surface proteins and decreasing the chain length for dissemination (16). A role for GelE in preventing biofilm formation has also been described by multiple research groups (17, 18).

Because E. faecalis has been implicated in various infections, including endocarditis, bacteremia, and urinary tract infections (19), GelE has been studied for its interaction with human proteins. A thorough characterization of GelE revealed multiple host cleavage targets, including glucagon and cholecystokinin, among several other substrates (12). More recently, GelE has been shown to cleave the C3-α chain of the human complement system, promoting immune evasion (20). Additionally, GelE can degrade the tight junction protein E-cadherin, contributing to intestinal inflammation and impaired barrier integrity (13). Our work adds GLP-1, among other metabolic factors, to the list of GelE targets.

Our data from Luminex assays using a panel of metabolic substrates show that GelE is able to degrade more substrates than was previously suspected, and furthermore, that GelE has an enhanced ability to degrade some substrates over others, including GLP-1, GIP, glucagon, and leptin. If occurring in the human body, degradation of these various substrates could have differing, sometimes contradictory, effects on host metabolic processes. GLP-1, GIP, and PYY are secreted basolaterally by enteroendocrine cells and have beneficial functions for host metabolism, and their absence would likely permit a change in glucose homeostasis, leading to hyperglycemia and an increase in food intake. The degradation of insulin, which is made and secreted in the pancreas, by GelE would likely result in similar hyperglycemia, and a reduction in pancreatic peptides might allow for increased food intake. Conversely, degradation of glucagon from alpha cells of the pancreas might result in hypoglycemia. Leptin is produced in adipocytes, and its degradation would likely cause dysregulation of fat accumulation and processing. Finally, MCP-1 and TNF-α are proinflammatory cytokines, and disruption of their signaling by degradation from GelE might delay an immune response during pathogen invasion. Importantly, whether these cleavage events are occurring and have relevant consequences in a whole biological system needs to be confirmed *in vivo* with comprehensive animal studies. Based on proximity, GelE crossing the epithelial layer would first encounter molecules secreted into the lamina propria, and so we suspect that GI hormones such as GLP-1, GIP, and PYY would be primary targets for degradation by GelE.

Our study supports the findings that E. faecalis and its protease GelE can compromise an epithelial layer and gain access to the basolateral environment (13–15). This is not surprising from a clinical perspective, as E. faecalis is implicated in cases of bacteremia and sepsis (19). For the topic of this study, this access could allow GelE to contact vital substrates responsible for host metabolic homeostasis as they are secreted into the GI lamina propria. Even mild inflammation often observed in individuals with metabolic syndrome, coupled with bacterial instigators like GelE from E. faecalis, could create a weakened epithelium (21). Once the integrity of the epithelium is damaged, luminal contents have an opportunity to move from the lumen of the GI tract and into the lamina propria just on the other side of the epithelial layer, where many hormones and metabolites are secreted before moving into the circulatory system. Furthermore, there is little indication regarding whether GelE could also be capable of diffusing into circulation as most hormones and nutrients do. Additionally, others have demonstrated by proof of concept that the microbiome encodes dipeptidyl peptidase-4 (DPP-4)-like activity that can traverse the epithelium and further propose that this activity is capable of modulating protein digestion and ultimately host metabolism and behavior (22); while DPP-4 and GelE work via different proteolytic mechanisms, the idea of bacterial proteases modulating host proteins and peptides is gaining traction.

Interestingly, members of the Enterococcus genus have been linked to obesity in children and adolescents, as well as to mice consuming a Western diet (23, 24). While not characterized in this study, we also identified several Clostridium isolates whose supernatants are capable of decreasing GLP-1 levels *in vitro*. We suspect that this activity is also the result of a secreted protease cleaving GLP-1, supporting the idea that the microbiome produces a suite of proteases capable of interfering with host metabolism. Although E. faecalis and *Clostridium* spp. have been implicated in infection, they can also behave as commensal organisms and often live inconspicuously in our GI tracts. It is important to understand all of the interactions of these organisms with their host and not just the overt pathogenic functions.

In summary, the results of this study demonstrate that GelE, a recognized virulence factor of E. faecalis, can degrade the human GI hormone GLP-1, among other metabolic substrates. The degradation of GLP-1 likely occurs by slight damage to the intestinal epithelium, allowing GelE to translocate across the epithelial layer and access GLP-1. While it would be reckless to assume that this activity is an etiology of metabolic disease, we do believe that interference with natural GLP-1 signaling by microbial degradation of GLP-1 could be a contributing factor to the development of disease. An important next step for this work is to assess the contribution of GelE to intestinal barrier permeability and the development of metabolic syndrome *in vivo*. Finally, this study adds a novel mechanism of action to the ever-growing list of host-microbe interactions.

## MATERIALS AND METHODS

### Bacterial strain isolation.

Bacterial strains for screening were isolated previously from fecal, breast milk, and ileum biopsy samples (8). Genetic mutant strains of Enterococcus faecalis (OG1RF, TX5266 [*fsrB* mutant], TX5264 [*gelE* mutant], TX5243 [*sprE* mutant], and TX5128 [*gelE sprE* mutant]) were generously gifted from the Danielle A. Garsin Laboratory (University of Texas, Health Science Center) (10). E. faecalis S613 was generously gifted from the Cesar A. Arias Laboratory (University of Texas, Heath Science Center) (11).

### Bacterial growth and preparation of cell supernatants.

Bacterial isolates were streaked from frozen glycerol stocks onto GM17 agar (M17 plus 0.5% [wt/vol] glucose) plates and incubated anaerobically overnight at 37°C. One colony was inoculated into 5 ml of GM17 broth and incubated overnight at 37°C, followed by one more subculture into GM17 broth and incubation overnight at 37°C. Once grown, bacterial cultures were centrifuged at 5,000 × *g* for 20 min. Supernatants were collected and lyophilized (FreeZone dryer; Labconco), followed by storage at –80°C until used for subsequent assays.

### 16S rRNA gene sequencing of isolates.

To identify the bacterial isolates, bacteria were streaked on GM17 agar plates from frozen glycerol stocks and incubated at 37°C for 24 to 48 h. The bacterial colony mass was then resuspended in 800 μl of sterile water, transferred to sterile bead-beating tubes, and homogenized for 2 min in a mini-beadbeater-96 (BioSpec Products). The tubes were centrifuged at 8,000 × *g* for 30 s, and the supernatants were used for 16S rRNA gene PCR amplification with Phusion high-fidelity DNA polymerase (New England BioLabs) in a 20-μl reaction mixture, according to the manufacturer’s protocol, using sequencing primers 8F and 1492R. The amplification cycle consisted of an initial denaturation at 98°C for 30 s, followed by 26 cycles of 10 s at 98°C, 20 s at 51°C, and 1 min at 72°C. Amplification was verified by agarose gel electrophoresis. For sample cleanup, DNA was treated with ExoSAP-IT reagent (Thermo Fisher) and incubated at 37°C for 15 min, followed by a 15-min incubation at 80°C to inactivate the enzyme. The product was cooled and sent to Genewiz for sequencing, according to company protocol. Upon return of the sequencing data, the sequences were compared to the NCBI BLAST database.

### Screening for GLP-1-stimulatory activity using NCI-H716 cells.

NCI-H716 (American Type Culture Collection [ATCC] CCL-251) cells were grown in RPMI (ATCC) medium supplemented with 10% (vol/vol) heat-inactivated newborn calf serum (NBCS; Gibco). Cultures were maintained at a concentration of 2 × 10^5^ to 8 × 10^5^ cells/ml and used at passages 15 to 40 for cell studies. For cell studies, 96-well plates were coated with 100 μl of 10 mg/ml Matrigel (BD Biosciences) for 2 h at room temperature. Following coating, NCI-H716 cells were seeded at a concentration of 1 × 10^5^ cells/well in Dulbecco’s modified Eagle’s medium (DMEM) supplemented with 10% (vol/vol) NBCS, as determined by trypan blue staining using a hemocytometer. Two days later, lyophilized bacterial supernatants were resuspended in Krebs buffer (Sigma) containing 0.2% (wt/vol) bovine serum albumin (BSA) and 0.03% (wt/vol) bovine bile and incubated on the NCI-H716 cells at 37°C with 5% CO_2_. 4-Phorbol 12 myristate 13-acetate (PMA, 2 μM) was used as a positive control, as it is a potent stimulator of GLP-1 secretion through the activation of protein kinase C (PKC). Following a 2-h incubation, supernatants were collected and analyzed for total GLP-1 levels by ELISA (MilliporeSigma), according to the manufacturer’s protocol. Cell viability was monitored using the PrestoBlue cell viability reagent (Thermo Fisher Scientific), following the manufacturer’s instructions.

### Characterization studies with TPEN, heat, and size.

For metalloprotease inhibitor studies, E. faecalis strains were subcultured 0.1% (vol/vol) from an overnight culture into GM17 containing the indicated concentration of *N,N,N′,N′*-tetrakis(2-pyridylmethyl)ethane-1,2-diamine (TPEN). Cultures were grown overnight, and supernatants were collected as described above and incubated with 500 pM GLP-1 (GenScript) for 4 h at room temperature, followed by storage at –80°C until ready for GLP-1 quantification by ELISA.

For heat treatment studies, bacterial supernatants were heated to 100°C for 30 min. Samples were then used in an NCI-H716 cell assay, as described above, followed by storage at –80°C until ready for GLP-1 quantification by ELISA.

For size fractionation studies, bacterial supernatants were separated by size using centrifugal filter units (Amicon) and centrifuged as described above. Samples were then used in an NCI-H716 cell assay as described above, followed by storage at –80°C until ready for GLP-1 quantification by ELISA.

### Protease knockout studies.

Supernatants from E. faecalis protease mutants and controls (OG1RF, TX5266 [*fsrB* mutant], TX5264 [*gelE* mutant], TX5243 [*sprE* mutant], and TX5128 [*gelE sprE* mutant]) were collected from an overnight culture grown in GM17 aerobically at 37°C by centrifugation, as described above. Supernatants were incubated with 500 pM GLP-1 (Tocris or GenScript) for 4 h at room temperature, followed by storage at –80°C until ready for GLP-1 quantification by ELISA, as described above.

### RNA collection and quantitation of *gelE* expression.

E. faecalis were subcultured at 1% (vol/vol) from an overnight culture into GM17. After 5 h of incubation, cells were collected by centrifugation, resuspended in RNA*later* solution (Invitrogen), and stored at –80°C. Cells were washed in 1× phosphate-buffered saline (PBS), resuspended in 1 ml RTL buffer (Qiagen RNeasy kit), and lysed by bead beating (2 × 1 min) at 4°C, followed by RNA extraction according to the manufacturer’s instructions. cDNA was synthesized using SuperScript III reverse transcriptase (Invitrogen), following the manufacturer’s recommended protocol. Quantitative PCRs were performed using Power SYBR green master mix (Applied Biosystems) with either E. faecalis 16S rRNA-specific (forward [f], 5′-CCGAGTGCTTGCACTCAATTGG-3′; reverse [r], 5′-CTCTTATGCCATGCGGCATAAAC-3′) or *gelE*-specific (f, 5′-CGGAACATACTGCCGGTTTAGA-3′; r, 5′-TGGATTAGATGCACCCGAAAT-3′) primers (25). The expression of *gelE* was normalized to that of the 16S rRNA, and the data were analyzed using the 2^−ΔΔ^*^CT^* method.

### Cell culture growth and assays of T84 monolayers.

Growth cultures and assays of T84 cells were performed by methods described previously, with slight modifications (26). T84 human colonic epithelial cells (ATCC CCL-248) were propagated in tissue culture-treated T75 flasks (Cellstar), as indicated by the ATCC. When between 90 and 100% confluence, T84 cells were treated with 0.25% trypsin and plated onto 24-well, 3.0-μm polycarbonate membrane transwell filters (catalog no. 3415; Costar) at a density of 8 × 10^4^ cells/well. The electrical resistance of the monolayer was monitored over the course of 2 to 3 weeks, and monolayers with a transepithelial electrical resistance (TEER; Millicell ERS-2; Millipore) of >800 Ω/cm^2^ were used for GLP-1 cleavage assays.

To prepare bacteria, on the day of the assay E. faecalis strains were subcultured 1% (vol/vol) from an overnight culture into GM17 and grown for 5 h, as described above. Whole-culture (cells plus supernatant) samples were diluted in DMEM–F-12 (Gibco) to a concentration of 1 × 10^7^ CFU/ml, and supernatant samples were diluted 50% in DMEM–F-12. All samples were neutralized to a pH of 6.8 to 7 using 3 M NaOH.

Once prepared, the TEER was measured for each monolayer, followed by 100 μl of E. faecalis sample added to the upper chamber of the transwell, and 500 μl of tissue culture medium containing 500 pM GLP-1 (GenScript) was added to the lower chamber. After a 16 h of incubation at 37°C in 5% CO_2_, 200 μl of medium from the lower chamber was removed and stored at –80°C until ready for GLP-1 quantification by ELISA, as described above. A final TEER measurement was taken for each monolayer.

### Luminex.

A Milliplex multiplex assay was performed using a Metabolic Luminex kit (MilliporeSigma) to measure total GLP-1, glucagon, gastric inhibitory peptide/glucose-dependent insulinotropic peptide (GIP), leptin, peptide YY (PYY), pancreatic peptide, insulin, monocyte chemoattractant protein-1 (MCP-1), interleukin-6 (IL-6), and tumor necrosis factor alpha (TNF-α). Bacterial supernatants were collected from an overnight culture by centrifugation as described above, diluted in bacterial culture medium as indicated, and incubated with recombinant protein (GenScript; Sigma, Tocris) of each analyte for 4 h at room temperature, followed by storage at –80°C until ready for analyte quantification. The Milliplex multiplex assay was run according to the protocol provided by the manufacturer.

### Statistical analysis.

Statistical analyses were performed using GraphPad Prism version 8.0 (San Diego, CA, USA). The experimental results are expressed as the means ± standard deviation. Statistical significance was set at a *P* value of <0.05. One-way statistical comparisons were carried out using one-way analysis of variance (ANOVA), followed by multiple comparisons of the means using Tukey’s *post hoc* analysis, for the GLP-1 secretion experiments in NCI-H716 cells, protease knockout mutation assay, and degradation preference of GelE using a Luminex assay (undiluted supernatants). Two-way ANOVA, followed by multiple comparisons of the means using Tukey’s *post hoc* analysis, was performed for the T84 cell experiments.
