# Supplementary figures and images for: Degradation of the Incretin Hormone Glucagon-Like Peptide-1 (GLP-1) by Enterococcus faecalis Metalloprotease GelE
Source: mSphere. 2020 Feb 12;5(1):e00585-19. doi: 10.1128/mSphere.00585-19 (PMC7021470; doi:10.1128/mSphere.00585-19)

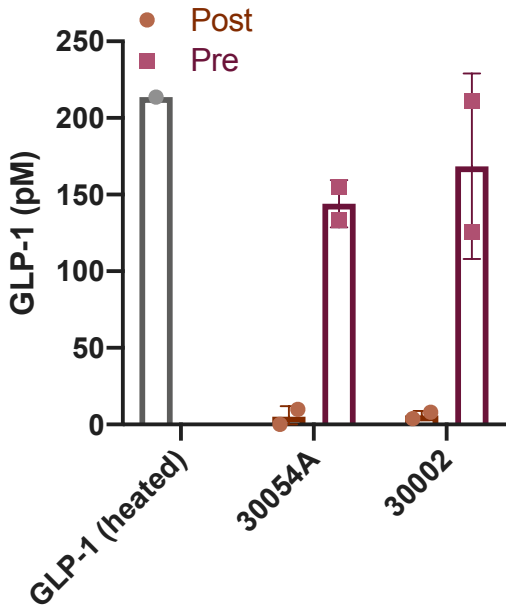

Supplement: FIG S4 [file mSphere.00585-19-sf004.pdf]
